# Bowel Function in Survivors of Rectal Cancer Managed with Watch-and-Wait Versus Surgery

**DOI:** 10.1007/s12029-025-01389-4

**Published:** 2026-01-26

**Authors:** Sina Vatandoust, Luigi Sposato, David Wattchow, John Leung, Amitesh Roy, Dayan de Fontgalland, Paul Hollington, Gang Chen, Shahid Ullah, Michael Z. Michael, Christos S. Karapetis

**Affiliations:** 1 Flinders Medical Centre, Bedford Park, Australia; 2 Flinders University, Bedford Park, Australia; 3 GenesisCare, Adelaide, Australia; 4 Cancer Health Services Research, University of Melbourne, Melbourne, Australia; 5 Centre for Health Services Research in Cancer, Peter MacCallum Cancer Centre, Melbourne, Australia

**Keywords:** Rectal cancer, Watch-and-wait, Sphincter-preserving surgery, Bowel function, Low anterior resection syndrome (LARS), Memorial sloan kettering bowel function instrument (MSK-BFI), Patient-reported outcomes

## Abstract

**Background:**

Sphincter-preserving surgery for rectal cancer is associated with long-term bowel dysfunction, yet data on outcomes in patients managed with watch-and-wait is limited. This study compared bowel function in rectal cancer survivors managed with watch-and-wait versus sphincter-preserving surgery and evaluated the performance of two instruments: the Memorial Sloan Kettering Bowel Function Instrument (MSK-BFI) and the Low Anterior Resection Syndrome (LARS) score within each cohort.

**Patients and Methods:**

In this cross-sectional study, rectal cancer survivors treated with long-course chemoradiotherapy between 2011 and 2019 at Flinders Medical Centre were recruited. Participants without stomas completed the MSK-BFI, LARS, and comorbidity questionnaires. Demographics and clinical data were obtained from medical records. Outcomes were compared between the sustained watch-and-wait (WW) and surgical (SURG) cohorts. Associations were examined using regression models. Instrument comparisons were performed in each cohort using factor analysis.

**Results:**

Of the 190 eligible survivors, 67 participants were included (WW, n=33; SURG, n=34). Surgery was associated with significantly worse bowel function across MSK-BFI domains and higher rates of Any LARS (91% vs. 67%) and Major LARS (70% vs. 36%). Female sex was independently associated with poorer bowel function. Factor analysis revealed that one factor accounted for bowel function in the SURG cohort, whereas two factors were required in the WW cohort, indicating greater complexity.

**Conclusion:**

Surgery and female sex are associated with significantly worse bowel function in rectal cancer survivors. The WW cohort demonstrated more complex symptom patterns, supporting concurrent use of MSK-BFI and LARS until a WW-specific tool is developed. A larger, prospective study is underway to investigate the findings further.

**Supplementary Information:**

The online version contains supplementary material available at 10.1007/s12029-025-01389-4.

## Micro Abstract

This cross-sectional study compared bowel function in rectal cancer survivors managed with watch-and-wait versus sphincter-preserving surgery. Surgery and female sex were associated with significantly worse bowel function. Factor analysis suggested that bowel dysfunction in the watch-and-wait cohort was more complex, supporting concurrent use of MSK-BFI and LARS until a watch-and-wait–specific tool is developed.

**Supplementary Information:**

The online version contains supplementary material available at 10.1007/s12029-025-01389-4.

## Clinical Practice Points

Sphincter-preserving surgery is associated with significantly worse bowel function compared with watch-and-wait.

Female sex is an independent risk factor for poorer bowel function among rectal cancer survivors.

In watch-and-wait survivors, bowel dysfunction is more complex and may require broader assessment.

Concurrent use of MSK-BFI and LARS is recommended in research and clinical practice until a tool validated for the watch-and-wait population is available.

These findings highlight the importance of tailored survivorship care and counselling in rectal cancer.

**Supplementary Information:**

The online version contains supplementary material available at 10.1007/s12029-025-01389-4.

## Introduction

Rectal cancer is a common malignancy. In the setting of locally advanced disease, pre-operative treatment followed by surgery has historically been the standard of care. Pre-operative therapies, also known as neoadjuvant therapies, include long-course chemo-radiotherapy (using a fluoropyrimidine chemotherapy agent) or short-course radiation, with or without additional chemotherapy. In 15–40% of cases (depending on the neoadjuvant approach), such treatments can lead to the disappearance of detectable cancer on clinical, endoscopic and radiologic assessments, which is referred to as a clinical complete response (cCR) [1, 2].

For patients achieving cCR, over the past decades, a watch-and-wait approach, pioneered by Brazilian researchers, has been proposed as an alternative to surgery [3]. Over time, this strategy has gained increasing recognition as a standard of care for selected patients with cCR. While accumulating evidence supports the safety of this approach [4], there is a growing need to understand its long-term functional outcomes, including bowel function.

Surgery for rectal cancer is linked to both short- and long-term consequences. Beyond the risks of perioperative complications and mortality (5), patients may experience enduring bowel dysfunction (6, 7) as well as challenges with urinary and sexual function (8). In many patients with low rectal cancers, surgery results in a permanent stoma. However, in those with mid- or upper-rectal tumours, sphincter-preserving procedures may be possible, avoiding the need for a permanent stoma.

Bowel dysfunction is well-documented following sphincter-preserving surgery [5]. However, there are limited data on bowel function in the expanding population of survivors managed with watch-and-wait and how they compare with survivors who have undergone sphincter-preserving surgery. Existing studies have been limited by small sample sizes [6, 7], less comprehensive assessment tools [8], or a lack of direct comparisons between watch-and-wait and surgical approaches [9, 10]. Moreover, currently available instruments, primarily designed to assess postoperative bowel function, have not been validated or systematically compared in non-operative cohorts.

In this study, we examined bowel function among rectal cancer survivors managed with sphincter-preserving surgery versus watch-and-wait, and evaluated associations between clinical variables, including surgery, and outcomes. We also compared two widely used instruments for assessing bowel function. As an exploratory, hypothesis-generating analysis, we further evaluated a small subgroup within the surgical cohort: participants who were initially managed non-operatively but subsequently underwent sphincter-preserving surgery for local regrowth, and compared them with participants who proceeded to standard sphincter-preserving surgery after completing chemoradiation.

## Methods

Study design and participants:

This cross-sectional study included adult survivors of non-metastatic rectal adenocarcinoma treated with long-course fluoropyrimidine-based chemoradiotherapy (50.4 Gy radiation combined with either fluorouracil or capecitabine) between 2011 and 2019 at Flinders Medical Centre, Adelaide, Australia. The chemoradiotherapy regimen used was similar across the study population and included a long-course, fluoropyrimidine-based chemoradiotherapy protocol, consisting of 50.4 Gy in 28 fractions [11] delivered concurrently with either continuous-infusion 5-fluorouracil [12] or oral capecitabine [13].

Participants completed study questionnaires either by mail or during in-person clinic visits. Health-related quality of life outcomes in this cohort have been reported separately [14]. The present analysis focuses specifically on bowel function and includes only participants without a stoma at the time of data collection.

Demographic data for all eligible patients were obtained from the institutional registry to evaluate potential response bias. Age and sex distributions were compared between respondents and non-respondents.

Instruments and data sources

### Instruments and Data Sources

Bowel function was assessed using the Memorial Sloan Kettering Bowel Function Instrument (MSK-BFI) [15] and the Low Anterior Resection Syndrome score (LARS score) [16]. The MSK-BFI is an 18-item questionnaire developed to assess bowel function after sphincter-preserving rectal surgery. It covers domains such as frequency, urgency/soilage, and dietary impact and generates a global score and a total score. The global score is calculated as the sum of these three subscales, providing an overall measure of bowel function. The total score comprises the global score plus independent items that are not part of the three subscales, offering a comprehensive measure of overall bowel function [7, 15]. These independent items are: incomplete evacuation, continence of gas, discrimination between gas and stool, and the need for another bowel movement within 15 min of the previous one. The scores are treated as continuous variables, with higher scores indicating better bowel function [15]. The LARS score is a brief 5-item instrument designed to evaluate bowel dysfunction following sphincter-preserving rectal resection, with a focus on incontinence, frequency, and urgency. Scores are categorised as No LARS, Minor LARS, or Major LARS, with higher scores reflecting worse bowel function [16]. Comorbidity burden was measured using the Self-Administered Comorbidity Questionnaire (SCQ). The SCQ is a validated instrument designed to assess comorbidity burden based on patient self-report. It captures the presence of common chronic conditions, associated treatments, and resulting limitations, providing a total score that reflects overall comorbidity. Higher scores indicate a greater comorbidity burden [17]. Demographics, tumour, and treatment data were collected from medical records.

### Cohort Definitions

At the time of data collection, participants were categorised by surgical history into two cohorts: those who had undergone sphincter-preserving surgery prior to data collection (SURG) and those managed non-operatively with a sustained watch-and-wait approach (WW).

Within the SURG cohort, we identified an exploratory subgroup comprising participants who initially underwent watch‑and‑wait but subsequently required sphincter‑preserving surgery for local regrowth (salvage surgery) and compared them descriptively with participants who proceeded to standard sphincter‑preserving surgery after completing chemoradiotherapy. These subgroup analyses were hypothesis‑generating only, with no adjustment for multiplicity and limited inferential interpretation.

### Ethics

The study was approved by the institutional Human Research Ethics Committee. All participants provided written informed consent.

### Statistical Analysis

We report the demographics and baseline characteristics using descriptive statistics. We used the Student’s T-Test, the Wilcoxon rank-sum test, Fisher’s exact test, Pearson correlation, Spearman correlation, linear regression, and multiple regression tests to examine associations between variables. To compare different bowel function instruments, we conducted exploratory factor analysis (EFA) of the MSK-BFI and LARS score in the SURG and WW cohorts. Suitability for factor analysis was evaluated with Bartlett’s test of sphericity and the Kaiser-Meyer-Olkin (KMO) measure. The number of factors was guided by parallel analysis. Then, we performed factor analysis on the data from both subgroups, using two factors and Oblimin rotation to characterise correlated dimensions. Analyses were performed in R version 4.4.3 (2025-02-28) [18] and RStudio (version 2024.12.1 + 563).

## Results

**Fig. 1:**
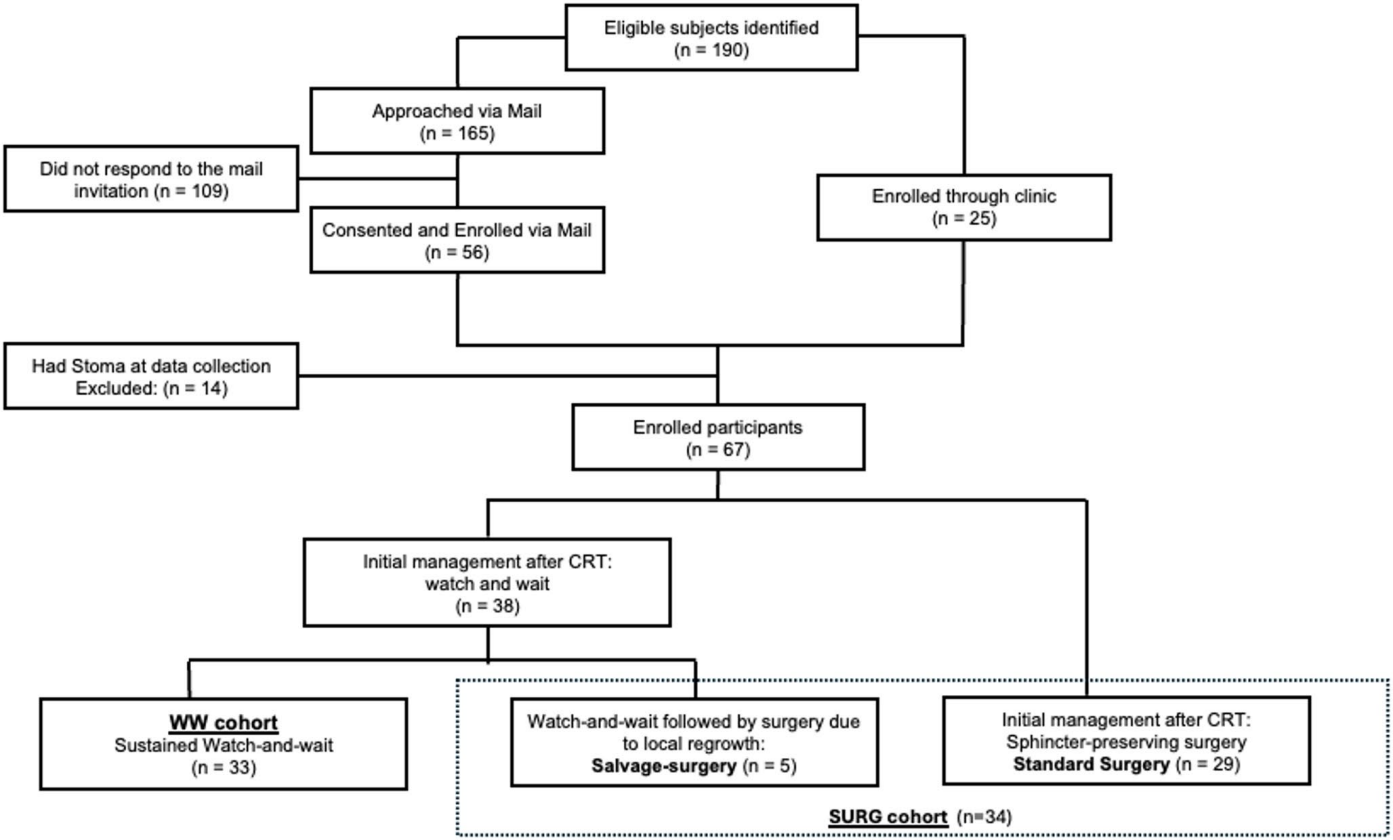
STROBE diagram: A total of 190 eligible rectal cancer survivors were identified. Of these, 165 were approached by mail and 25 through clinics, resulting in 67 enrolled participants after exclusions. At data collection, 33 participants were managed with sustained watch-and-wait, 29 with standard sphincter-preserving surgery, and 5 with salvage surgery after initial watch-and-wait. Fourteen participants with a stoma at the time of data collection were excluded.

Of 190 eligible survivors contacted, 81 consented to participate. Of these, 67 participants without stomas at the time of data collection were included in the analyses for this study. At the time of data collection, 33 (49.3%) were in the WW cohort and 34 (50.7%) in the SURG cohort. Within the SURG cohort, 5 participants (14.7%) were initially managed non-operatively but later underwent sphincter‑preserving surgery for local regrowth. The other 29 participants (85.3%) underwent standard sphincter‑preserving surgery after neoadjuvant treatment. Among participants invited by mail, median age did not differ significantly between respondents and non-respondents (*p* = 0.26), and sex distribution was also similar between groups (*p* = 0.98). Figure 1 depicts the STROBE diagram.

### Basic Characteristics

Baseline characteristics are presented in Table 1 for the overall study population as well as for each study cohort.

Table 1:

**Table 1:** Baseline characteristics of the whole study population and by management cohort (surgery [SURG] vs. watch-and-wait [WW]). Values are presented as median (interquartile range, IQR) unless otherwise specified. Categorical variables are reported as a number (percentage). SCQ = Self-Administered comorbidity Questionnaire; BMI = body mass index

Characteristic	Whole group (*N* = 67)	SURG cohort (*N* = 34)	WW Cohort (*N* = 33)
Median age at diagnosis (IQR) – y	65 (60–72)	62 (58–68)	70 (63–76)
Median age at data collection (IQR) – y	69 (64–75)	68 (62–71)	72 (64–80)
Female sex no. (%)	22 (32.8)	11 (32.4)	11 (33.3)
Time from diagnosis to data collection (IQR) – months	39 (18–65)	59 (21–82)	28 (18–45)
Comorbidity burden: Median SCQ score (IQR)	4.0 (2.0–6.50)	3.0 (1.0–5.0)	5.0 (2.0–8.0)
BMI: Median (IQR)	28.7 (25.9–32.5)	28.9 (25.3–32.3)	28.0 (26.3–32.9)
Median tumour distance from anal verge (IQR) (cm)	6.5 (4.2–8.4)	7.0 (5.0–8.6)	5.4 (3.8–8.0)
Disease stage at diagnosis no. (%) Stage I Stage II Stage III Stage IV (resected metastatic disease)	8 (11.9%) 17 (25.4%) 41 (61.2%) 1 (1.5%)	5 (14.7%) 8 (23.5%) 20 (58.8%) 1 (2.9%)	3 (9.1%) 9 (27.8%) 21 (63.6%) 0 (0%)

Age at diagnosis of rectal cancer was lower in the SURG cohort than in the WW cohort (*p* < 0.01), but age at data collection did not differ (*p* > 0.05). Disease stage at diagnosis was not different between the two cohorts (*p* > 0.05). Time since diagnosis was longer in the SURG cohort (*p* < 0.05). The WW cohort had a higher comorbidity burden (*p* < 0.05). The tumour location was lower (closer to the anal verge) in the WW cohort (*p* < 0.05).

### MSK-BFI Correlations

Age at data collection showed a weak positive correlation with the MSK-BFI diet subscale (higher score indicating better function) (*r* = 0.25, *p* < 0.05). Clustering score (having another bowel movement within 15 min of the last movement) (higher score indicating better function) showed a moderate positive correlation with age at diagnosis (*r* = 0.35, *p* < 0.01), a weak positive correlation with age at data collection (*r* = 0.29, *p* < 0.05) and a weak negative correlation with time since diagnosis (−0.28, *p* < 0.05).

All the other aspects of MSK-BFI (global score, total score, subscales other than diet, and individual items other than clustering) did not correlate with age at data collection, time since diagnosis, comorbidity burden, BMI or tumour distance from the anal verge (all *p* > 0.05).

### LARS Correlations

In the study population, Age at data collection showed a weak negative correlation with the presence of Major LARS (*r* = − 0.29, *p* < 0.05), indicating that younger patients were more likely to report Major LARS. There was a weak positive correlation between tumour distance and the presence of Major LARS (higher tumours (more distance) were associated with a slightly higher chance of Major LARS) (*r* = 0.25, *p* < 0.05). Reporting Any LARS (Minor or Major LARS) was not correlated with age at data collection, time since diagnosis, comorbidity score, BMI or tumour distance. Time since diagnosis, BMI, and comorbidity burden were not correlated with the presence of Major or Any LARS (all *p* > 0.05).

### Sex Differences

**Fig. 2:**
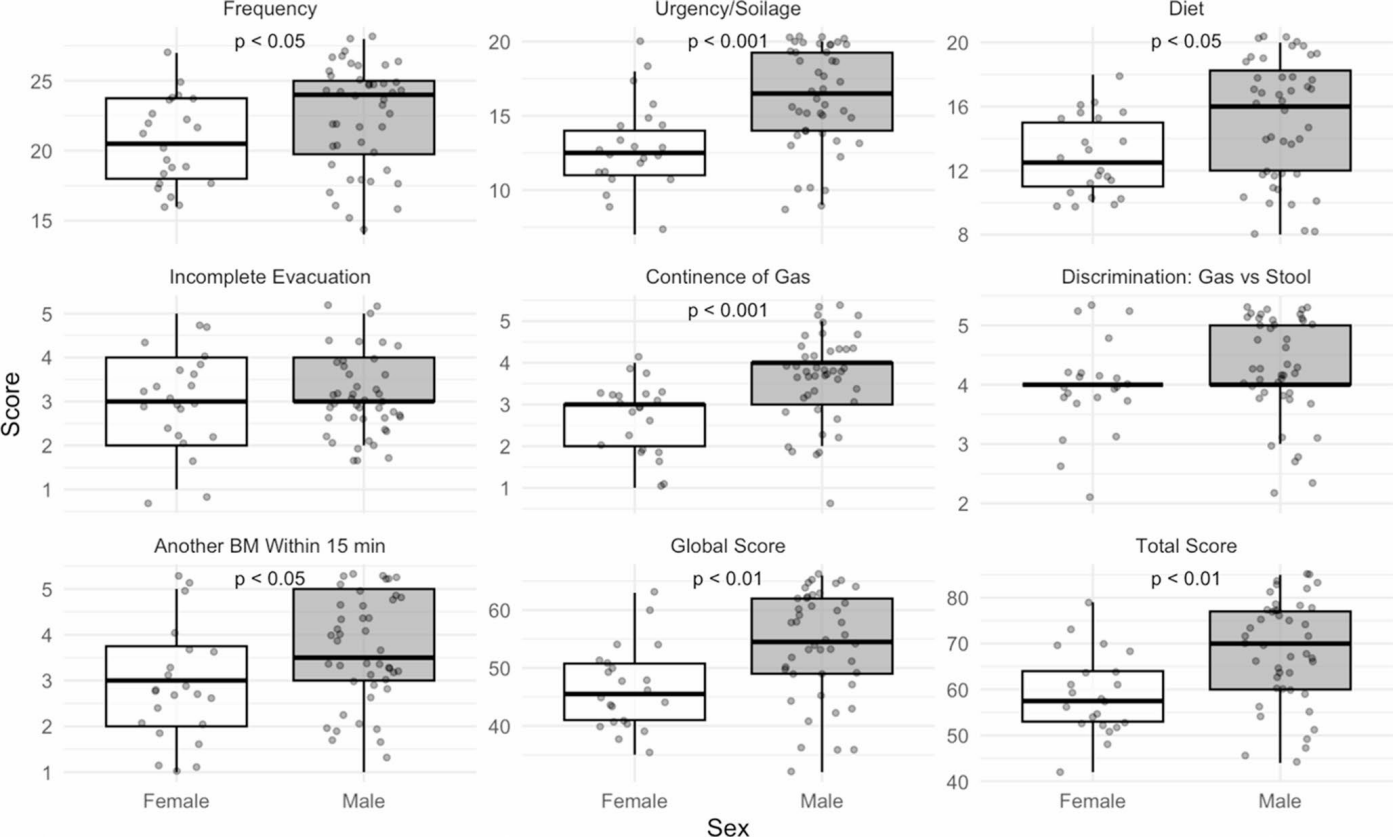
Comparison of MSK-BFI Bowel Function Inventory Scores by Sex. Female sex depicted in white and Male sex in grey colour. A lower score represents worse bowel function. ‘Another BM Within 15 min’: another bowel movement within 15 min of the last bowel movement. P-values are presented when statistically significant

MSK-BFI: Female participants, compared to male participants, reported lower MSK BFI global and total scores (worse function) as well as lower scores in all MSK BFI subscales and items (all *p* < 0.05) except for the Incomplete Evacuation item and the Discrimination: Gas vs. Stool item. (Figure 2)

LARS score: 14 male participants (31.8%) reported scores in keeping with No LARS, while all female participants reported a degree of LARS (Minor or Major). Female participants were more likely to report Major LARS compared to male participants [OR = 10.7 (95% CI: 2.6–65.0), *p* < 0.001].

### Effect of Sphincter-Preserving Surgery

#### MSK-BFI

**Fig. 3:**
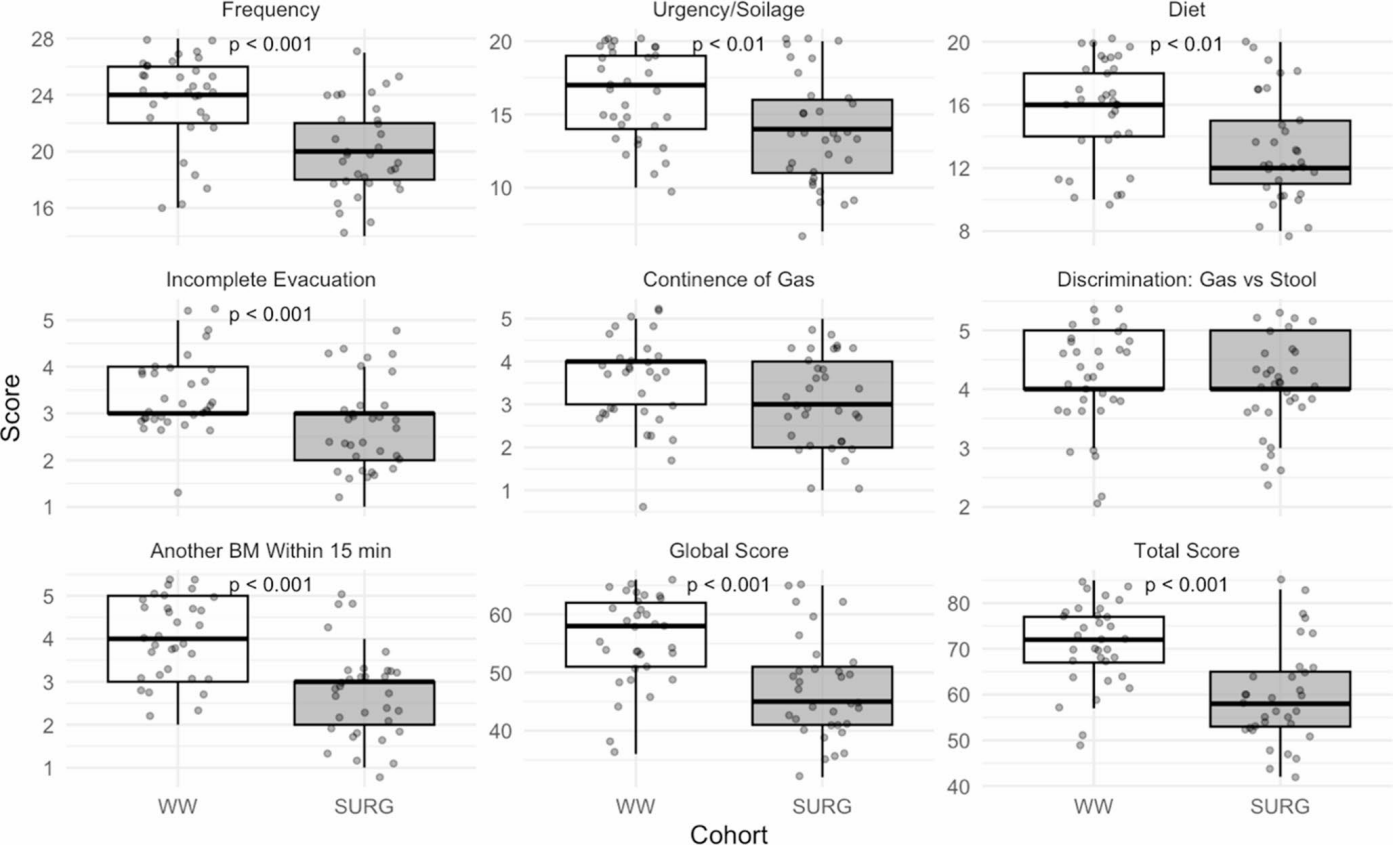
Comparison of MSK-BFI scores between the sustained watch-and-wait (WW) and surgery (SURG) cohorts (in white and grey colours respectively). Lower scores indicate worse bowel function. Another BM Within 15 min refers to an additional bowel movement occurring within 15 min of the previous one. P-values are shown where statistically significant

Surgery was associated with worse MSK-BFI outcomes, including lower global and total scores and impairment across most domains, specifically frequency, urgency, diet, incomplete evacuation, and the need for a second bowel movement shortly after the first. (Figure 3)

In a multiple linear regression model, surgery was significantly associated with lower MSK-BFI total scores (β = − 11.27, *p* < 0.001), indicating worse bowel function compared to non-surgical management. Female sex was also independently associated with lower scores (β = − 9.41, *p* < 0.001), in keeping with worse bowel function in female participants. The model explained 38% of the variance in bowel function scores (adjusted R² = 0.38). Adding variables such as age, time since diagnosis, tumour distance, comorbidity score, and BMI did not improve model performance, and none of these covariates were statistically significant.

#### LARS Scores

Any LARS: participants in the SURG cohort were significantly more likely to experience Any LARS (Minor or Major) compared with the WW cohort (91% vs. 67%; *p* < 0.05, OR = 4.9; 95% CI, 1.1–30.5). Major LARS: This was also significantly more common in the SURG cohort compared with the WW cohort (70% vs. 36%; *p* < 0.05, OR = 3.9; 95% CI, 1.3–12.8). The odds ratio for sex could not be reliably estimated in the model for Any LARS due to the absence of participants without LARS in the female subgroup, resulting in complete separation and model instability.

Multivariable logistic regression, Any LARS: In a multivariable model, the SURG cohort was significantly associated with increased odds of experiencing Any LARS (OR = 6.3; 95% CI, 1.6–32.8; *p* < 0.05). Other covariates, including age, time since diagnosis, comorbidity score, BMI, and tumour distance, were not statistically significant. Since all female participants reported either Minor or Major LARS, the effect of sex could not be assessed in this model due to complete separation.

Multivariable logistic regression, Major LARS: In a model including surgery, sex, and tumour distance, greater tumour distance was associated with higher odds of Major LARS (*p* < 0.05), contrary to clinical expectations. Tumour distance showed a weak positive correlation with surgery (*r* = 0.27, *p* < 0.05); therefore, it was residualised to account for this. In the adjusted model, the SURG cohort (OR = 8.7; 95% CI, 2.3–42.5; *p* < 0.01), female sex (OR = 29.5; 95% CI, 5.9–233; *p* < 0.001), and residualised tumour distance (per cm) (OR = 1.3; 95% CI, 1.05–1.68; *p* < 0.05) were all independently associated with higher odds of Major LARS.

#### Instrument Comparison: LARS vs. MSK-BFI

Bartlett’s test of sphericity was significant for both cohorts (SURG: χ² = 61.6, *p* < 0.001; WW: χ² = 133.8, *p* < 0.001), indicating that the correlation matrices were suitable for factor analysis. The KMO statistic was 0.71 for SURG and 0.76 for WW, indicating acceptable sampling adequacy. In the SURG cohort, parallel analysis supported a single-factor solution that accounted for 43.7% of the variance; MSK-BFI items (frequency, urgency/soilage, and continence of gas) loaded strongly on this factor, whereas LARS items showed mixed or weaker loadings. In the WW cohort, parallel analysis supported a two-factor solution that explained 42.7% of the variance, with LARS items loading primarily on the first factor and MSK-BFI items related to urgency and diet loading on the second.

## Non-Operative Management Followed by Sphincter-Preserving Surgery due to Regrowth

Among participants initially managed with a non-operatively who later underwent sphincter-preserving surgery for local regrowth (salvage-surgery, *N* = 5), there was no statistically significant difference in MSK-BFI domain or global scores, nor the prevalence of Any LARS or Major LARS, compared with participants who proceeded directly to standard sphincter-preserving surgery after chemoradiation (standard-surgery, *N* = 29). Given the small sample, estimates are imprecise. Further results are presented in the Supplementary files.

## Discussion

The study population is representative of the contemporary population of patients with rectal cancer managed either with surgery or with a watch-and-wait approach. The study cohorts were comparable across most demographic factors and tumour characteristics. Both cohorts received an identical long-course, fluoropyrimidine-based chemoradiotherapy regimen delivered in accordance with available guidelines [11–13]. This uniform neoadjuvant approach minimises potential confounding related to treatment heterogeneity and supports the validity of the functional comparisons observed between the WW and SURG groups. The consistent chemoradiotherapy regimen also facilitates comparison with international series, where similar dosing schedules and concurrent fluoropyrimidine use are standard practice. While broadly comparable, there were a couple of differences between the two cohorts. Firstly, the time since diagnosis was longer in the surgical group; this is explained by the fact that non-operative management is a relatively novel approach and has only been accepted as a standard of care over the past few years. Importantly, time since diagnosis was not significantly associated with bowel function. In addition, the WW cohort had a higher comorbidity burden and more distal tumours, which are considerations in selecting patients for non-operative management by many experts [19]. The higher comorbidity burden observed in the WW cohort may partly reflect clinical decision-making, as patients with greater medical complexity are often considered less suitable for major pelvic surgery. Tumour height may also have influenced initial management, as lower tumours are associated with lower sphincter-preservation rates and a higher likelihood of permanent stoma formation. These covariates were accounted for in the analyses, strengthening the robustness of our findings.

Although the survey response rate was modest, comparison of demographic characteristics showed no significant differences in age or sex between respondents and non-respondents, indicating that systematic response bias is unlikely for these variables.

## Surgery and Bowel Function

Surgery was consistently associated with worse bowel function across the MSK-BFI and LARS instruments and participants who had undergone surgery reported poorer outcomes across multiple domains of bowel function. These findings were evident in univariate comparisons and multivariable models, highlighting the significant functional consequences of sphincter-preserving surgery in survivors of rectal cancer.

Both the WW and SURG cohorts received identical neoadjuvant chemoradiotherapy; therefore, any differences in bowel function are most plausibly attributed to the effects of surgery rather than variations in radiation or chemotherapy. To explain these differences, it is important to consider the physiological basis of post-surgical bowel dysfunction. The precise mechanisms underlying post-surgical bowel dysfunction remain incompletely defined but are widely recognised to be multifactorial, reflecting an interplay between structural, motility-related, and neurogenic factors [20]. Surgical resection alters pelvic anatomy and disrupts the rectal reservoir and evacuation mechanisms required for continence. The loss or reduction of rectal capacity, distortion of the rectoanal angle, and trauma to the anal sphincter complex or its innervation can each compromise coordinated storage and defecation [21, 22]. The neorectum that forms after resection often exhibits abnormal compliance and hyperactive motility [23, 24]. Moreover, denervation of extrinsic autonomic pathways can impair rectosigmoid coordination [25, 26], while disruption of the intrinsic enteric nervous system further limits compensatory storage through dysregulated colonic peristalsis [27]. Taken together, these alterations align with the symptom complex captured by LARS.

Accordingly, patients managed with a watch-and-wait strategy retain the rectal reservoir, sphincteric apparatus, and intact pelvic innervation, preserving the physiological mechanisms that maintain continence and controlled evacuation. This structural and functional preservation likely explains the superior bowel outcomes observed in the WW cohort, despite exposure to identical chemoradiotherapy regimens.

Furthermore, the MSK-BFI scores and the prevalence of major LARS in the SURG cohort were similar to previous reports in the literature [7, 8]. This similarity supports the assumption that the surgical subgroup in our study is broadly representative of rectal cancer survivors treated with sphincter-preserving surgery.

## Sex Differences in Bowel Function

There are conflicting reports in the literature regarding the association between sex and bowel dysfunction in rectal cancer survivors. While some studies have indicated that bowel dysfunction is more prevalent in female survivors of rectal cancer [28], some have suggested male sex as a risk factor [29]. However, others have not found a significant association between LARS and sex (male or female) [30]. Interestingly, in a study of low anterior resection syndrome in a reference population (participants without a history of colorectal cancer), Major LARS was significantly more prevalent in women than men [31]. In our study, female participants reported worse bowel function than males across both bowel function instruments used. All female participants reported LARS (either Minor or Major), and MSK-BFI scores were lower across all domains. These results suggest that sex is an important and under-recognised factor contributing to bowel dysfunction after rectal cancer treatment. This finding will also help recognise populations of survivors who are at higher risk of developing significant bowel symptoms, prompting early planning and tailored survivorship care.

## Tumour Distance and LARS

Increased tumour distance from the anal verge was associated with worse bowel outcomes, which may in part reflect the correlation between tumour location and treatment selection, as higher tumours are less likely to be considered for non-operative management. However, this correlation was weak, and tumour distance was residualised to account for its shared variance with surgery. In the adjusted model, residualised tumour distance retained a modest independent association with the presence of Major LARS. This finding may represent variance in tumour height unrelated to surgical selection, potentially reflecting individual anatomical differences, or stochastic variability within a modest sample. Previous studies have generally reported a negative association between anastomotic height and LARS severity [30]; thus, our observation may represent a context-specific pattern that warrants confirmation in larger, prospectively stratified studies.

## LARS Score and MSK-BFI Factor Analysis

Factor analysis of the two bowel function instruments indicated that for the SURG cohort, one factor may be sufficient to capture the primary underlying bowel function issues, focusing on urgency, frequency, and continence of gas, as seen with MSK-BFI. This is in keeping with data from the study by Quezada-Diaz et al., who also suggested that in survivors of rectal cancer managed with sphincter-preserving surgery, MSK-BFI and LARS scores are correlated and have similar validity [32]; however, the mentioned study did not include non-operative patients. In our study, the WW cohort demonstrated the need for two factors to capture the bowel function symptoms, suggesting that the bowel function issues in this group are more complex and require a broader assessment. In this group, the MSK-BFI captured aspects not fully covered by the LARS tool, as LARS score primarily assessed urgency and continence. To our knowledge, the two instruments have not been directly compared and evaluated within a watch-and-wait population previously.

Based on our results and given the absence of a tool specifically designed for the watch-and-wait population, it is prudent to use the MSK-BFI and LARS instruments concurrently to enable a more comprehensive evaluation of bowel function in this group. In contrast, patients in the surgery group may be adequately assessed with the MSK-BFI alone. However, given the small sample size in each cohort, these findings should be regarded as exploratory and require validation in larger datasets.

## Non-Operative Management followed by Sphincter-Preserving Surgery due to Regrowth

Within the SURG cohort, we did not observe a marked difference in bowel function between the salvage-surgery subgroup and the standard-surgery subgroup, which is cautiously reassuring given concerns that delayed surgery might impair function. Nonetheless, due to small sample size, the analysis is underpowered and vulnerable to heterogeneity in assessment timing and operative details, as well as residual confounding and selection bias. Accordingly, these results should be viewed as hypothesis-generating and require confirmation in larger, prospectively designed studies.

### Limitations

The study has several limitations. First, the sample size was modest, which limited power for subgroup analyses and increased the risk of a type II error. Second, the retrospective design may introduce bias, particularly in participant selection and recall. Third, there were inherent differences between the WW and surgical groups, including time since diagnosis and comorbidity burden, which could influence functional outcomes and may not be fully accounted for despite statistical adjustments.

Despite these limitations, the study remains one of the few to evaluate long-term bowel function in patients managed with watchful waiting compared to surgery. Other available studies have not accounted for variables such as comorbidities. Furthermore, to our knowledge, this is the first study to assess the role of different bowel function instruments and compare them in WW survivors. A larger prospective study is underway to allow for better matching between groups, minimise bias, and validate these findings [33]. Nevertheless, the current analysis provides important real-world insights into the functional outcomes of non-operative management in rectal cancer.

## Conclusion

This study highlights that surgery and the female sex significantly affect bowel function in rectal cancer survivors. Surgery is associated with worse bowel function, including increased urgency, frequency, and dietary restrictions. Female survivors also report poorer bowel function compared to males, emphasising the need to consider this factor in the survivorship spectrum. The results suggest that a different approach is needed to assess bowel function in the WW cohort, highlighting the need for a bowel function assessment instrument specifically designed for WW and, in the meantime, supports the use of both the LARS score and MSK-BFI in this group to cover the complex bowel dysfunction issues in this group of survivors. Finally, within the SURG cohort, bowel function did not differ markedly between salvage- and standard-surgery subgroups, a cautiously reassuring but exploratory finding that warrants further confirmation in larger studies.

## Supplementary Information

Below is the link to the electronic supplementary material.

ESM1 Supplementary Material 1 (DOCX 164 KB)

## Data Availability

No datasets were generated or analysed during the current study.
