# Supplementary material for: Bowel Function in Survivors of Rectal Cancer Managed with Watch-and-Wait Versus Surgery
Source: J Gastrointest Cancer. 2026 Jan 26;57(1):26. doi: 10.1007/s12029-025-01389-4 (PMC12835014; doi:10.1007/s12029-025-01389-4)
Supplement: Supplementary file 1 — Supplementary Material 1 (DOCX 164 KB) [file 12029_2025_1389_MOESM1_ESM.docx]

**Supplementary files:**

Supplementary Figure:


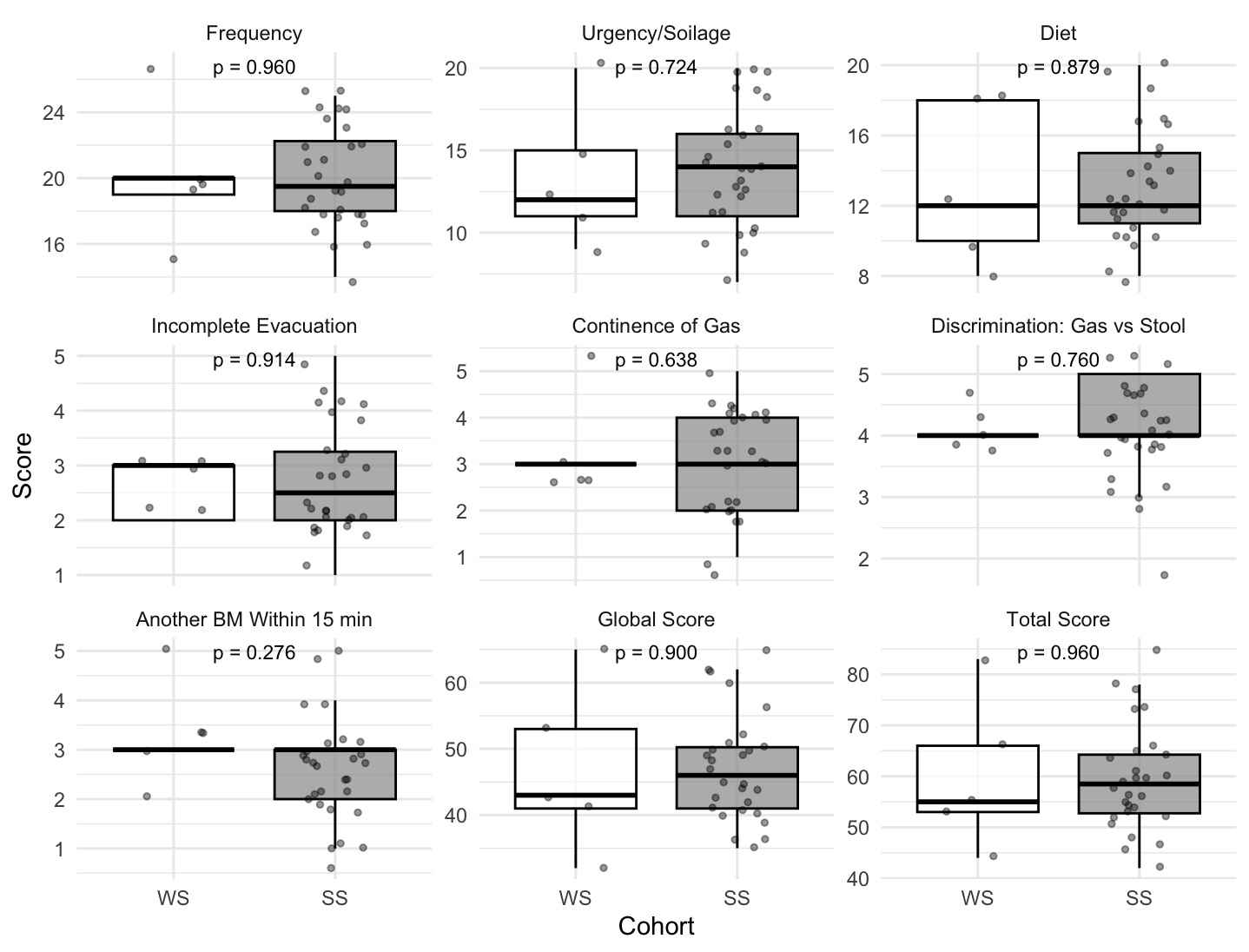


Supplementary Figure: Comparison of MSK-BFI Bowel Function Inventory Scores in Surgical Subgroups. Salvage surgery subgroup (WS) depicted in white and Standard Surgery (SS) in grey colour. A lower score represents worse bowel function. ‘Another BM Within 15 min’: another bowel movement within 15 minutes of the last bowel movement. P-values are presented. Given the small sample size, this analysis should be considered exploratory.
